# Health-related quality of life in Chinese medical staff: a latent profile analysis

**DOI:** 10.3389/fpubh.2023.1183483

**Published:** 2023-05-05

**Authors:** Jing Huang, Jianing Zhu, Yifan Ruan, Haitao Zhou, Wenjian Guo, Aishu Dong

**Affiliations:** ^1^Department of Cardiology, The Second Affiliated Hospital of Wenzhou Medical University, Wenzhou, China; ^2^Department of Radiotherapy and Oncology, The Second Affiliated Hospital of Wenzhou Medical University, Wenzhou, China

**Keywords:** health-related quality of life, China, medical staff, latent profile analysis, COVID-19

## Abstract

**Objective:**

To investigate subgroups of health-related quality of life (HRQoL) in the Chinese medical staff and identify the demographic factors associated with these profiles.

**Methods:**

574 Chinese medical staff were surveyed online. HRQoL was measured by using the 36-Item Short Form Health Survey, Version 2. Latent profile analysis (LPA) was used to identify the profiles of HRQoL. The associations between HRQoL profiles and covariates were assessed using multinomial logistic regression.

**Results:**

Three HRQoL profiles were developed: low HRQoL at 15.6%, moderate HRQoL at 46.9%, and high HRQoL at 37.6%. Multinomial logistic regression showed night shift times, aerobic exercise conditioning, and personality type significantly predicted the profile membership.

**Conclusion:**

Our findings develop earlier approaches that only used total scores to evaluate this group’s HRQoL and help them with tailored interventions to promote better HRQoL.

## 1. Introduction

As defined by the World Health Organization (WHO), the quality of life (QoL) concerns an individual’s current situation and their morals, expectations, beliefs, and concerns—all of which are influenced by the complex relationship between an individual’s physical health, mental health, social relationships, and environmental change (1–3). Health-related quality of life (HRQoL) mainly covers the health-related parts of QoL. In health economics, HRQoL is usually measured using a scale and serves as a barometer of personal, community, and socioeconomic development (4). The interest in HRQoL of medical staff has shown an increasing trend in recent years, along with the recognition of its impact on public health and medical development. The higher scores of HRQoL, the better general health and the fewer disorders or disabilities (5).

The nature of the work of healthcare professionals is both stressful and challenging, which can pose a threat to them and may impact their HRQoL (6, 7). In addition, the specific working environment and interpersonal relationships can impact the HRQoL of medical personnel (8). Studies have proved that medical staff was more likely to be stressed (6, 9), anxious, and depressed (10–12), especially since the outbreak of COVID-19. A meta-analysis also discovered consistent evidence for the widespread and profound impact of large outbreaks on the mental health of frontline medical staff (13). Furthermore, medical staff is more likely to suffer from job burnout due to the enormous workload they experience in the workplace (14–16).

Further research supports the idea that the HRQoL of medical staff maintains and promotes their compassion and empathy for patients and is closely related to the quality of care they provide (17, 18). Consequently, understanding and measuring HRQoL among medical staff has recently emerged as a priority. Prevention of negative emotional and physical problems in health care workers and promotion of overall health is one of the priorities advocated by the state and public health authorities (19). Positive psychology holds that emphasizing a person’s vitality and virtues is crucial. It promotes personal and social development by leveraging inner and constructive strengths. The ultimate goal of positive psychology is to pursue human happiness (20). However, most current studies on this particular group have focused on describing negative emotions such as anxiety, depression, and job burnout rather than overall HRQoL.

Currently, a variety of scales were used to measure human beings’ HRQoL. The appropriate HRQoL measurement metrics, however, lack a standardized definition. The 36-Item Short Form Health Survey, Version 2 (SF-36 v2) has been used to measure this outcome (both mental health and physical health) and has its advantages (21, 22). It has been considered an appropriate tool for measuring HRQoL in different populations and is favored in terms of its psychometric properties and convenience for monitoring HRQoL (23, 24). Moreover, it also helps monitor HRQoL in the healthy population (25). Furthermore, most of the previous literature used composite scores on scales to assess the level of HRQoL of the medical staff, few studies used the SF-36v2 scale to measure (26, 27). Additionally, this approach does not allow for further population classification; it only offers a comprehensive assessment of the population’s HRQoL. In addition, quantitative research on different HRQoL profiles in medical staff has received scant attention in the literature.

A method focused on the individual called latent profile analysis (LPA) uses continuous variables to divide samples into more meaningful subgroups based on similar characteristics (28). LPA is also a statistical method for determining whether heterogeneous subgroups exist within a population of interest. It can determine the underlying characteristics of individuals based on their response patterns to explicit topics to understand the characteristics of people with different profiles (29, 30). It is helpful to learn more about the population characteristics of different potential profiles by using LPA to explore HRQoL in medical staff and identify the sociodemographic correlates to the profiles of QoL. At present, there are few potential profile models of the HRQoL of medical staff. In conclusion, more LAP-based research needs to be investigated.

Therefore, the main goal of this study was to find the distinct profiles of HRQoL in Chinese medical staff using an LPA approach. Then, we studied the sociodemographics associated with profile membership. This study will provide fundamental evidence for public health to create targeted intervention strategies to improve HRQoL in medical staff.

## 2. Method

### 2.1. Study design

A multi-center cross-sectional study was carried out. And the Strengthening the Reporting of Observational Studies in Epidemiology (STROBE) guidelines were followed in this study.

### 2.2. Participants

An online survey was conducted by enrolling a convenience sample of medical staff mainly from six hospitals (the 1st Affiliated Hospital of Wenzhou Medical University, the 2nd Affiliated Hospital of Wenzhou Medical University, the 2nd Hospital of Dalian Medical University, the 2nd Affiliated Hospital of Zhongguo Medical University, Lishui People’s Hospital, and Chenzhou 3rd People’s Hospital) in China. Medical staff was eligible if they had legal rights and obligations to the hospital. Medical staff was excluded if they were unwilling to participate in the study or had severe mental health problems. Two were disqualified from the total 574 eligible participants who agreed to take part because their responses were not complete—resulting in a 572 (99.7%) valid sample size.

### 2.3. Data collection

Between May and July 2020, online questionnaires were collected anonymously from medical staff who met the criteria. Every Internet Protocol (IP) address was only permitted to access the survey once in order to avoid double enrollment. Each questionnaire took about 15–20 min to complete. The system would exclude questionnaires that took less than 10 min to complete.

### 2.4. Measures

#### 2.4.1. Sociodemographic characteristics

The characteristics of medical staff included gender, age, marital status, education level, professional title, department, position, employment status, work time per day, night shift times per week, individual income monthly, exercise condition and self-reported personality.

#### 2.4.2. Health-related quality of life

The SF-36v2 was used to assess self-perceived HRQoL. The eight dimensions of physical functions (PF), role-physical (RP), physical pain (PP), health in general (HG), vitality (VT), social functions (SF), role-emotional (RE), and mental health (MH) accounted for 10 items, 4 items, 2 items, 5 items, 4 items, 2 items, 3 items, and 5 items, respectively, for a total of 36 items. The physical component summary (PCS) and the mental component summary (MCS) are the two subscales among the eight dimensions. The present study showed satisfactory reliability (Cronbach’s *α* = 0.861).

### 2.5. Ethical consideration

The study followed the ethical principles of the Helsinki declaration (31). The institution the corresponding author was associated with approved our study through its institutional review board (LCKY2019-288). Before formally starting to fill out the questionnaire, participants will be shown a statement about informed consent, the purpose of the study, and the content of the study. Participants have the right to decide whether or not to continue filling out the questionnaire. All patients gave informed consent.

### 2.6. Statistical analysis

The following versions of softwares were used to analyze the data: Mplus version 8.3 (Muthen & Muthen, Los Angeles, CA, Unites States), SPSS version 25.0 (IBM, Armonk, NY, Unites States), and Stata version 14.1 (StataCorp LP, 1985–2015). All variables were first subjected to descriptive statistics. Second, different LPA models were developed to explore the profiles of HRQoL among 572 medical staff. The Akaike information criterion (AIC), Bayesian information criterion (BIC), adjusted Bayesian information criterion (aBIC), and the Lo–Mendell–Rubin adjusted likelihood ratio test (LMRT) were used to determine the ideal number of profiles (32). Lower values for the AIC, BIC, and aBIC signify a better-fitting model. Models with different numbers of latent profiles were compared using LMRT. When a k-class model showed a non-significant value, a k-1 class model should be accepted. Entropy was used to evaluate the classification precision of the model, varying between 0 and 1, with larger values being better. When the number exceeds 0.80, the classification accuracy has been determined to be adequate. Item means were examined using Analysis of Variance (ANOVA) to determine if profiles derived from LPA differed significantly. Third, when the best profile model was determined, each profile was named according to its distributions. The Chi-squared test was used to determine how the various profiles differed in terms of sociodemographic traits, and Bonferroni method for multiple comparisons. Multinomial logistic regression was used to adjust confounding factors. All variables with univariate *p* values <0.05 were chosen as independent variables for the multinomial regression models.

## 3. Results

### 3.1. Descriptive statistics

There were 521 female participants (91.1%) and 51 male participants (8.9%). Nurses and doctors accounted for 474 (82.9%) and 59 (10.3%), respectively. The mean age of the medical staff was 34.18 ± 6.36 (range 21–55). The majority of participants said they had a college degree (73.8%, *n* = 422) or higher in education (11.2%, *n* = 64), were married (81.8%, *n* = 464), with primary (44.2%, *n* = 253) or medium (46.0%, *n* = 263) professional title, and were officially employed by the hospital (68.2%, *n* = 390). More than half participants reported working more than 8 h a day (65.2%, *n* = 373) and one night shift per week (50.0%, *n* = 286). Individual participants’ monthly incomes were as follows: 237 (41.4%) reported having an income of 6,000 RMB or less, 143 (25.0%) reported having an income between 6,000 RMB and 8,000 RMB, and 192 (33.6%) reported having an income of >8,000 RMB. Most participants never exercised (43.2%, *n* = 247) or exercised 1–2 h per day (41.8%, *n* = 239). As for personality, approximately 24.1% (*n* = 138) participants considered themselves extroverted, 22.9% (*n* = 131) considered themselves introverted, and 54.5% (*n* = 303) considered themselves intermediate. Table 1 displayed all of the remaining general data.

**Table 1:** Comparison of socio-demographic characteristics among different health-related quality of life (HRQoL) profiles (*n* = 572).

Variables		Total sample	Low HRQoL	Moderate HRQoL	High HRQoL	*χ* ^2^	*p*
Gender						4.255	0.119
	Male	51(8.9)	4(4.5)	22(8.2)	25(11.6)		
	Female	521(91.1)	85(95.5)	246(91.8)	190(88.4)		
Age						3.486	0.480
	20–29	142(24.8)	22(24.7)	67(25.0)	53(24.7)		
	30–39	324(56.6)	55(61.8)	154(57.5)	115(53.5)		
	≥40	106(18.5)	12(13.5)	47(17.5)	47(21.9)		
Marital status						1.603	0.822
	Single	99(17.3)	19(21.3)	46(17.2)	34(15.8)		
	Married	464(81.8)	69(77.5)	218(81.3)	177(82.3)		
	Divorce	9(1.6)	1(1.1)	4(1.5)	4(1.9)		
Education						2.138	0.710
	Below college	86(15.0)	12(13.5)	39(14.6)	35(16.3)		
	College degree	422(73.8)	70(78.8)	199(74.3)	153(71.2)		
	Above college	64(11.2)	7(7.9)	30(11.2)	27(12.6)		
Professional title						3.304	0.508
	Primary	253 (44.2)	44(49.4)	120(44.8)	89(41.4)		
	Medium	263(46.0)	40(44.9)	119(44.4)	104(48.4)		
	High	56(9.8)	5(5.6)	29(10.8)	22(10.2)		
Department						16.990	0.386
	Medical	89(15.6)	22(24.7)	41(15.3)	26(12.1)		
	Surgical	61(10.7)	7(7.9)	33(12.3)	21(9.8)		
	Pediatric	30(5.2)	4(4.5)	14(5.2)	12(5.6)		
	Obstetrics and Gynecology	41(7.2)	6(6.7)	15(5.6)	20(9.3)		
	Emergency room	111(19.4)	14(15.7)	47(17.5)	50(23.3)		
	Operating room	18(3.1)	2(2.2)	10(3.7)	6(2.8)		
	ICU	26(4.5)	2(2.2)	16(6.0)	8 (3.7)		
	NICU	14(2.4)	3(3.4)	7(2.6)	4(1.9)		
	Others	182(31.8)	29(32.6)	85(31.7)	68(31.6)		
Position						4.063	0.397
	Doctor	59(10.3)	7(7.9)	29(10.8)	23(10.7)		
	Nurse	474(82.9)	79(88.8)	222(82.8)	173(80.5)		
	Others	39(6.8)	3(3.4)	17(6.3)	19(8.8)		
Employment status						0.890	0.641
	Official	390(68.2)	57(64.0)	186(69.4)	147(68.4)		
	Contract/temporary	182(31.8)	32(36.0)	82(30.6)	68(31.6)		
Work time per day						7.012	0.030
	<8	199(34.8)	25(28.1)	85(31.7)	89(41.4)		
	≥8	373(65.2)	64(71.9)	183(68.3)	126(58.6)		
Night shift times per week						11.238	0.024
	0	147(25.7)	16(18.0)	60(22.4)	71(33.0)		
	1	286(50.0)	46(51.7)	143(53.4)	97(45.1)		
	≥2	139(24.3)	27(30.3)	65(24.3)	47(21.9)		
Income per month						7.518	0.111
	<6,000	237(41.4)	45(50.6)	113(42.2)	79(36.7)		
	6,000–8,000	143(25.0)	18(20.2)	73(27.2)	52(24.2)		
	>8,000	192(33.6)	26(29.2)	82(30.6)	84(39.1)		
Exercise						17.023	0.002
	Never	247(43.2)	49(55.1)	121(45.1)	77(35.8)		
	1-2 h/week	239(41.8)	34(38.2)	113(42.2)	92(42.8)		
	≥3 h/week	86(15.0)	6(6.7)	34(12.7)	46(21.4)		
Personality						12.060	0.017
	Extroverted	138(24.1)	18(20.2)	57(21.3)	63(29.3)		
	Introverted	131(22.9)	30(33.7)	63(23.5)	38(17.7)		
	Intermediate	303(54.5)	41(46.1)	148(55.2)	114(53.0)		

Data presented as frequency (percentage).

Table 2 displayed the descriptive statistics and correlations of the grouping variables used. The highest mean was for BP, and the lowest mean was for VT, with little difference between the means of the eight subgroup variables. High positive correlations between RP and RE, GH and VT, GH and MH, VT and MH, SF and RE, and SF and RH were found in the results of the correlation analysis.

**Table 2:** Descriptive statistics and correlations.

Variables	M	SD	PF	RP	BP	GH	VT	SF	RE
PF	47.60	9.38	1						
RP	47.04	10.78	0.414***	1					
BP	47.79	9.25	0.372***	0.353***	1				
GH	44.51	9.28	0.356***	0.269***	0.441***	1			
VT	40.35	10.94	0.357***	0.276***	0.418***	0.628***	1		
SF	44.62	11.04	0.362***	0.461***	0.482***	0.434***	0.493***	1	
RE	44.83	12.85	0.376***	0.667***	0.332***	0.321***	0.417***	0.544***	1
MH	40.43	10.97	0.319***	0.290***	0.422***	0.581***	0.777***	0.589***	0.487***

M, mean; SD, standard deviation; PF, physical functioning; RP, role-physical; BP, bodily pain; GH, general health; VT, vitality; SF, social functioning; RE, role-emotional; MH, mental health.

*** *P*<0.001.

### 3.2. Latent profile analysis

**Figure 1:**
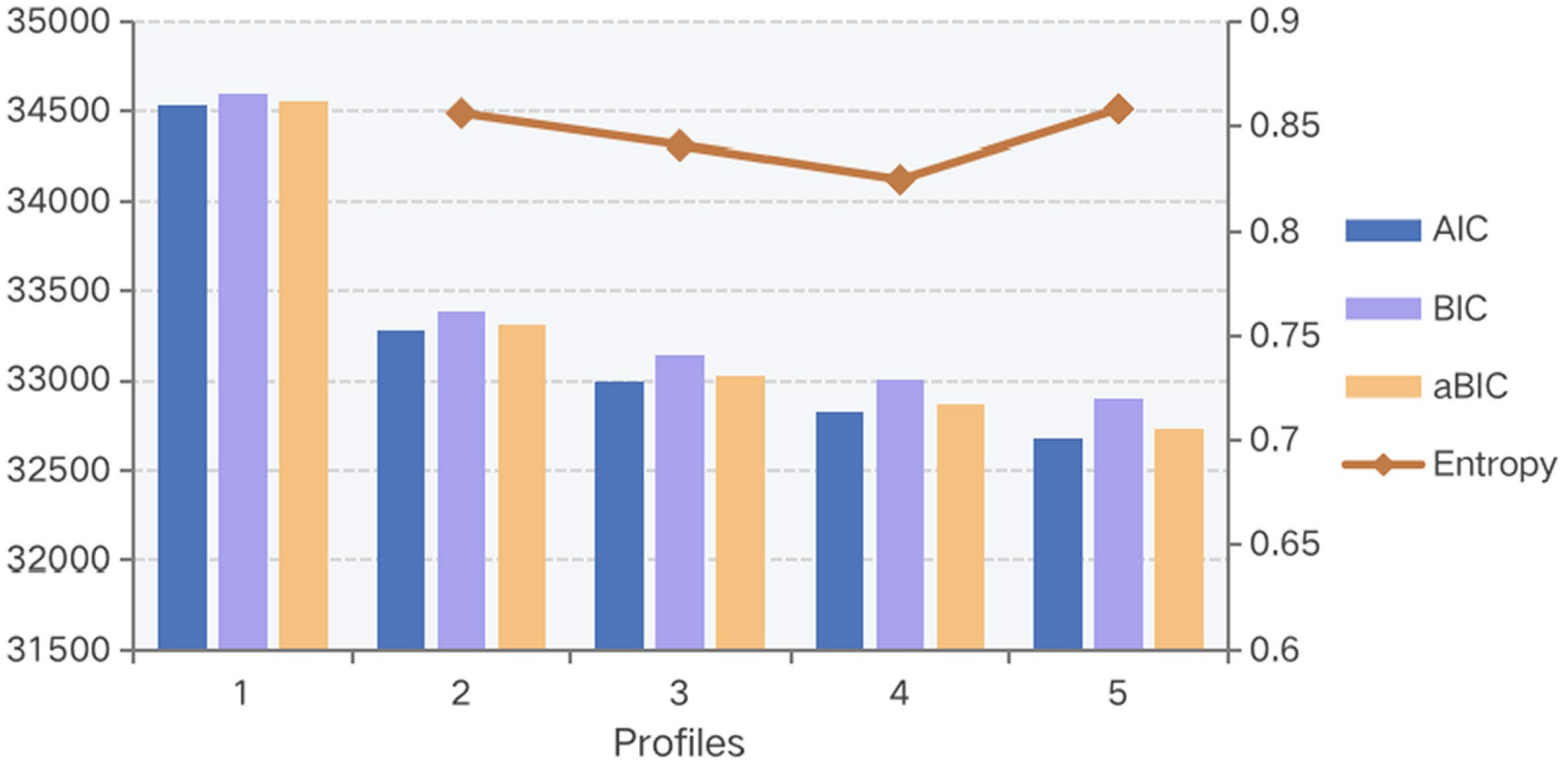
Comparison of the information criteria.

A one-to-five classification was present, according to information-based fit indices (Table 3). Figure 1 showed that AIC, BIC, and aBIC gradually improved (number decreased) as the number of profiles increased, and it displayed the trend of entropy. Considering both LMRT and BLRT showed significance in profiles 2 and 3. Based on the analysis of the available data, the three-profile model was selected for this study (33). It had a suitable entropy value of 0.841, indicating a distinct classification. The mean posterior probabilities that participants pertained to the latent profile where they were assigned were 91.6–95.0%, indicating that the three-profile models were credible (Table 4).

**Table 3:** Fit statistics for the latent class model with 1–5 classes.

	AIC	BIC	aBIC	Entropy	LMRT	BLRT	Latent class probability
					*p* value	*p* value	
1 class	34530.769	34600.355	34549.562				
2 class	33281.312	33390.040	33310.676	0.856	<0.001	<0.001	0.46329/0.53671
3 class	32991.884	33139.755	33031.820	0.841	0.0213	<0.001	0.15559/0.46853/0.37587
4 class	32821.123	33008.136	32871.630	0.825	0.0452	<0.001	0.13811/0.25000/0.24825/0.36364
5 class	32673.388	32899.543	32734.466	0.858	0.0972	<0.001	0.25175/0.01923/0.26573/0.11014/0.35315

AIC, Akaike information criteria; BIC, Bayesian information criteria; aBIC, adjusted Bayesian information criterion.

LMRT, Lo–Mendell–Rubin likelihood ratio test; BLRT, Bootstrapped likelihood ratio test.

**Table 4:** Average latent class probabilities for most likely latent class membership (Row) by latent class (Column).

	C1	C2	C3
C1	0.924	0.076	0.000
C2	0.041	0.916	0.043
C3	0.000	0.050	0.950

**Figure 2:**
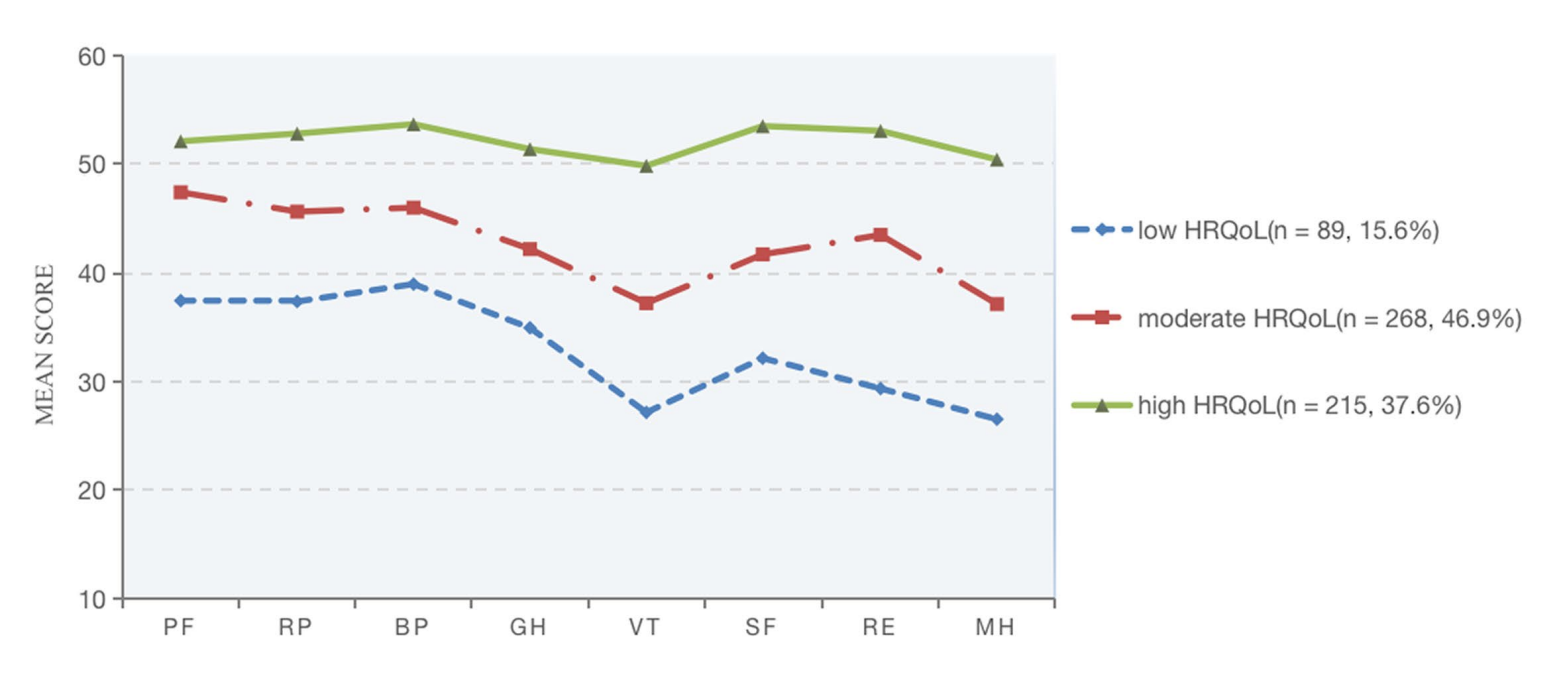
Item means for the three-profile mode of HRQoL. PF, physical functioning; RP, role-physical; BP, bodily pain; GH, general health; VT, vitality; SF, social functioning; RE, role-emotional; MH, mental health.

**Figure 3:**
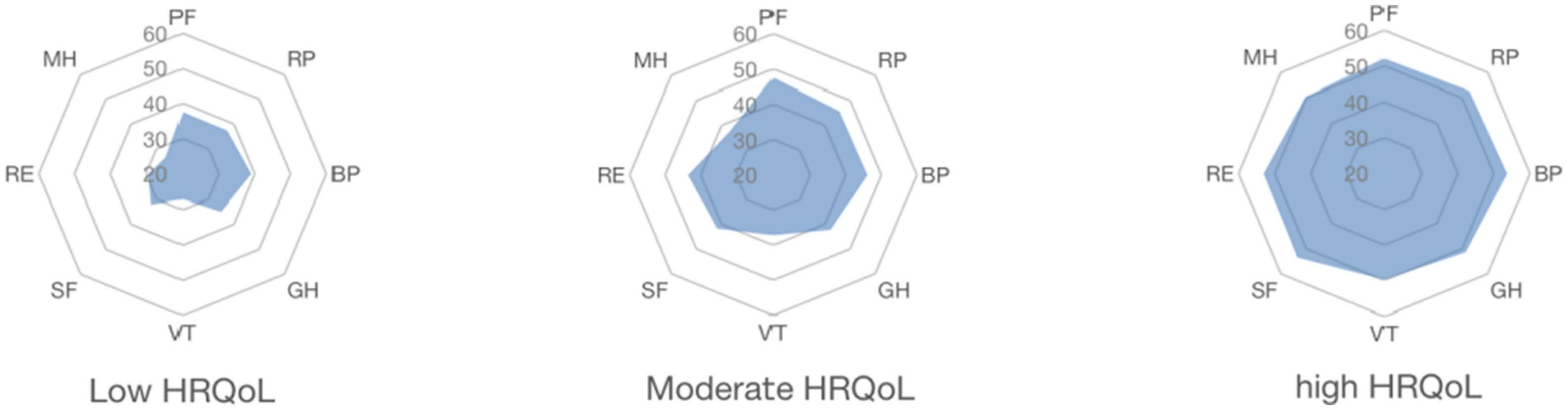
The tendencies of three distinctive HRQoL. PF, physical functioning; RP, role-physical; BP, bodily pain; GH, general health; VT, vitality; SF, social functioning; RE, role-emotional; MH, mental health.

Following that, the names of the three profiles were determined by their characterized patterns of HRQoL. And named C1–C3 as “Low HRQoL,” “Moderate HRQoL,” and “High HRQoL” individually, as seen in Figure 2 and Table 5. Significant mean differences between the manifest indicators for each profile were revealed by the three-profile solution (Table 5). The high HRQoL profile made up the second most common proportion of participants (*n* = 215, 37.6%). The moderate HRQoL profile was the largest profile (*n* = 268, 46.9%). The low HRQoL profile was the third most prevalent profile (*n* = 89, 15.6%). The tendencies of the three HRQoL profiles were illustrated in Figure 3.

**Table 5:** Descriptive statistics for health-related quality of life (HRQoL) disaggregated by latent profile class.

Variable	Class 1 Low HRQoL *n* = 89, 15.6%		Class 2 Moderate HRQoL *n* = 268, 46.9%		Class 3 High HRQoL *n* = 215, 37.6%		*F*
	M	SE	M	SE	M	SE	
PF	37.483	1.802	47.506	0.818	52.095	0.387	63.608***
RP	37.404	1.627	45.717	1.146	52.806	0.649	87.574***
BP	39.015	0.990	46.083	0.921	53.672	0.507	58.409***
GH	34.973	1.601	42.255	0.582	51.374	0.657	51.051***
VT	27.146	2.592	37.251	0.621	49.827	0.712	52.697***
SF	32.162	1.243	41.763	1.049	53.502	0.604	62.869***
RE	29.354	1.671	43.566	1.466	53.059	0.633	99.544***
MH	26.517	1.936	37.170	0.793	50.409	0.626	46.226***

*** *p* < 0.001. PF, physical functioning; RP, role-physical; BP, bodily pain; GH, general health; VT, vitality; SF, social functioning; RE, role-emotional; MH, mental health.

### 3.3. Predictor of latent profile membership

The Chi-squared test results revealed significant differences in work time per day (*χ*^2^ = 7.012, *p* = 0.030), night shift times per week (*χ*^2^ = 11.238, *p* = 0.024), exercise (*χ*^2^ = 17.023, *p* = 0.002), and personality (*χ*^2^ = 12.060, *p* < 0.017) between the three profiles (Table 1).

With the low HRQoL group as the reference group, multinomial logistic regression was further used to investigate the sociodemographic predictors of profile membership, and significant influencing factors in the Chi-square test were included (Table 6). In comparison to the low HRQoL group, medical staff with introverted traits (OR: 0.495; CI: 0.268, 0.915) had lower odds of being in the high HRQoL group. Compared to those who exercise more than 3 h per week, medical staff who never exercise (OR: 0.250; CI: 0.098, 0.638) had lower odds of being in the high HRQoL group than the high HRQoL group. Moreover, medical staff with no night shift per week (OR: 2.299; CI: 1.089, 4.855) were more likely to fall into the high HRQoL group than the low HRQoL group.

**Table 6:** Odds ratios for the covariates predicting latent profile membership.

Covariate		Moderate HRQoL vs. Low HRQoL			High HRQoL vs. Low HRQoL		
		OR	95%CI	*P*	OR	95%CI	*P*
Worktime							
	<8	0.940	0.544–1.625	0.824	0.651	0.371–1.143	0.135
	≥8	Ref	Ref	Ref	Ref	Ref	Ref
Night shift times per week							
	0	1.568	0.757–3.247	0.226	2.299	1.089–4.855	0.029
	1	1.333	0.749–2.373	0.328	1.176	0.635–2.179	0.606
	≥2	Ref	Ref	Ref	Ref	Ref	Ref
Exercise							
	never	0.469	0.183–1.197	0.113	0.250	0.098–0.638	0.004
	1-2 h/week	0.610	0.235–1.582	0.309	0.400	0.155–1.032	0.058
	≥3 h/week	Ref	Ref	Ref	Ref	Ref	Ref
Personality							
	Extroverted	0.854	0.452–1.614	0.627	1.183	0.620–2.258	0.610
	Introverted	0.603	0.344–1.059	0.078	0.495	0.268–0.915	0.025
	Intermediate	Ref	Ref	Ref	Ref	Ref	Ref

OR, odds ratio; CI, confidence interval. Low HRQoL as a refer.

## 4. Discussion

By using LPA analysis, three profiles representing the level of HRQoL of Chinese medical staff were created for the present study. They were low HRQoL, moderate HRQoL, and high HRQoL, which accounted for 15.6, 46.9, and 37.6%, respectively. According to our findings, the majority of participants were in the moderate HRQoL group and exhibited comparatively moderate levels of physical functions, role-physical, physical pain, general health, vitality, social functions, role-emotional, and mental health. Overall, it is important to value and improve the level of HRQoL among medical staff.

Medical staff in the low HRQoL profile represented the smallest percentage of the overall sample in the present study. Focusing on each dimension indicates that the average score for MH was only about 26 in the low HRQoL group, which is a large gap from the scores of the high HRQoL group (higher than 50), showing a bipartition trend. This suggests a potential need to tailor interventions to medical staff’s mental health condition. The study by Liu et al. (34) can support our view that of the 1,090 Chinese medical professionals, 13.3, 18.4, and 23.9% suffered from anxiety, depression, or both, respectively. Since the questionnaire was collected during the COVID-19 epidemic, those who were unprepared for a sudden outbreak are likely to experience mental health issues, especially the special population of medical staff, who dealt with greater challenges and stress (35). In conclusion, our study demonstrates the importance of mental health in improving the HRQoL of medical professionals. So among the various aspects of HRQoL, mental health needs the most attention in the present study.

It is worth noting that the third dimension-BP, had the highest mean score among all three profiles of HRQoL, followed by the second dimension-RP. This could be a result of the fact that, in comparison to other jobs, medical professionals will adopt more healthy behaviors, such as giving up smoking and drinking alcohol, putting more emphasis on eating well, and exercising more as they become more knowledgeable about diseases and health-related issues. Previous studies have also confirmed this view. A study by Liu et al. (36) revealed that healthcare workers are more concerned about food safety following a pandemic outbreak. A meta-analysis also suggested that interventions aimed at lifestyle change are more likely to improve nurses’ HRQoL (37). In other words, healthcare professionals who prioritize their own health will adopt healthier habits and lifestyles, enhancing their physical well-being, lessening physical discomfort, and ultimately enhancing their general HRQoL accordingly.

Multiple regression showed in the current research that factors influencing the HRQoL of medical staff included the work time per day and the nightshifts per week, respectively. The absence of nightshifts was a protective factor for the HRQoL of medical staff. In addition, we found that VT was the second lowest dimension in the three HRQoL profiles. The specific 4 items for this dimension are “Did you feel full of pep?” “Did you have a lot of energy?” “Did you feel worn out?” and “Did you feel tired?” The findings mentioned above imply that a lower VT score may be linked to long working hours, high intensity, stress brought on by frequently working nights, physical overdraft, and burnout. It’s consistent with the study by Silva et al. (38). Among medical staff, the dimensions with the lowest mean scores in the SF-36 were VT. A study from Italy also found that night nurses had lower HRQoL in all dimensions than the general population (39). As a result, by altering scheduling practices, better allocating human resources, and changing management paradigms, healthcare administrators can enhance the HRQoL for healthcare workers. It is important to note that a previous study (40) identified effort-reward imbalance as a significant factor contributing to work stress and fatigue in medical staff. Based on Siegrist’s effort-reward imbalance model (ERI) (41), the subsequent study can further explore the relevant factors influencing the psychological dimensions of this population. By the way, even though the univariate analysis demonstrated that work time per day was statistically significant, it was excluded from the logistic regression model. It probably due to the sample size is not large enough. Future studies could include a larger sample of medical staff for analysis.

This study also noted that the HRQoL of medical staff was related to exercise condition and personality type. As in previous studies (42, 43), medical personnel with exercise habits were more likely to maintain higher levels of mental well-being and physical health. Heidke et al. (44) found that physical inactivity was negatively related to HRQoL. These results support the statement in the present study that a higher frequency of weekly exercise is a protective factor for the HRQoL of medical personnel. Therefore, medical professionals can increase their HRQoL by increasing weekly exercise frequency. In addition, extroverts were more likely absent of depression and had high mental well-being (45). Consistent with previous research, individuals with introverted personalities were more likely to be in the low HRQoL group than those with intermediate personalities in the present study. Hence, introversion may be a risk factor for the HRQoL of medical staff. This suggests a potential need to tailor interventions according to personality differences.

A series of our findings can help provide medical staff with interventions for improving HRQoL. As HRQoL is sometimes used interchangeably with mental well-being. The majority of the interventions available today for healthcare professionals focus on psychological issues like anxiety and depression and how to control them. There are relatively few interventions that specifically address how to improve the quality of life of healthcare professionals. Consequently, it is imperative to develop interventions that take into consideration the relevant characteristics and cultural identity of Chinese medical personnel. Considering the findings of our study, future research might focus on enhancing the physical condition of medical personnel. Despite the fact that most medical professionals are knowledgeable about health issues, the intense work demands and lack of personal time present some challenges for this group in managing their own health. Psychological interventions can be used to alleviate symptoms like fatigue. For instance, mindfulness reduces the emotional burden on the nurse, which in turn reduces the level of burnout (46). And resilience training also showed a positive effect on medical staff’s anxiety and stress (47). However, psychological interventions typically need to be carried out over a longer period of time and by qualified psychologists. Our vision can be placed on the availability of direct and simple physical interventions to alleviate their fatigue and tiredness levels. This has immediate implications for medical professionals.

### 4.1. Strengths and limitations

Some contributions stem from the present study. The present findings reveal heterogeneity in the healthcare worker sample, implying the need for suitable quality of life improvement programs for various healthcare groups. As far as we are aware, there have not been any studies using LPA to investigate the variables affecting healthcare workers’ HRQoL. We can use the profile membership information provided by LPA to identify groups of healthcare professionals with various HRQoL traits. Integrating medical staff’s HRQoL traits with demographic characteristics in subsequent studies to find more targeted intervention plans. However, certain limitations should be taken into account. On the one hand, we cannot determine how profiles may change or stabilize over time or how profiles would predict the long-term HRQoL of medical staff due to the cross-sectional design of the current study. Further longitudinal studies can be performed to gather information on how medical staff’s HRQoL evolves over time. On the other hand, in the absence of objective assessment criteria, the level of HRQoL is self-reported results. Recall bias may affect study results. A multi-information strategy would be advantageous for upcoming research. What’s more, the extrapolation of the results may be somewhat constrained because the current study only focused on the population of Chinese healthcare workers. Nevertheless, convenience sampling can be unreliable and limit the generalization of research findings to other population groups; we consider to incorporate design-based principles such as randomization or systematic sampling into future survey designs.

## 5. Conclusion

The current study explored different profiles of HRQoL among Chinese medical staff to analyze the level of HRQoL of this population more specifically and we finally found three different levels of HRQoL traits among Chinese medical staff. The results of our study are important to the development of public health today.

## Data availability statement

The raw data supporting the conclusions of this article will be made available by the authors, without undue reservation.

## Ethics statement

The study was conducted according to the guidelines of the Declaration of Helsinki, and approved by the Research Ethics Committee of the Second Affiliated Hospital of Wenzhou Medical University (LCKY2019-288). Informed consent was obtained from all subjects involved in the study.

## Author contributions

JH and JZ wrote the main manuscript text. YR, HZ, and WG contributed to the data collection. JH contributed to the data analysis. AD contributed to the study design and had full access to all the data in the study, taking responsibility for the data analysis’s accuracy and the data’s integrity, and contributing to revising the article and final approval. All authors contributed to the article and approved the submitted version.

## Funding

This study was supported by the Medical Science and Technology Project of Zhejiang province, China (2022KY898) and the Science and Technology Plan Project of Wenzhou, China (Y2019038) provided funding for this study, which had no role in the study design, collection, analysis, or interpretation of the data, writing the manuscript, or the decision to submit the paper for publication.

## Conflict of interest

The authors declare that the research was conducted in the absence of any commercial or financial relationships that could be construed as a potential conflict of interest.

## Publisher’s note

All claims expressed in this article are solely those of the authors and do not necessarily represent those of their affiliated organizations, or those of the publisher, the editors and the reviewers. Any product that may be evaluated in this article, or claim that may be made by its manufacturer, is not guaranteed or endorsed by the publisher.
